# Acupuncture for the treatment of severe acute pain in Herpes Zoster: results of a nested, open-label, randomized trial in the VZV Pain Study

**DOI:** 10.1186/1472-6882-11-46

**Published:** 2011-06-05

**Authors:** Tamara Ursini, Monica Tontodonati, Lamberto Manzoli, Ennio Polilli, Cristina Rebuzzi, Gabriele Congedo, Sonia Di Profio, Patrizia Marani Toro, Augusta Consorte, Giuseppina Placido, Stefano Laganà, Claudio D'Amario, Carla Granchelli, Giustino Parruti, Lucio Pippa

**Affiliations:** 1 Infectious Diseases Unit, Pescara General Hospital, Pescara, Italy; 2 Section of Epidemiology and Public Health, University of Chieti, Italy; 3 Pain Management Clinic, Pescara General Hospital, Pescara, Italy; 4 Fondazione Onlus Camillo de Lellis per l'Innovazione e la Ricerca in Medicina, Pescara, Italy; 5 Pescara Health District, Pescara, Italy

## Abstract

**Background:**

Data on the potential efficacy of acupuncture (AC) in controlling intense or very intense pain in patients with Herpes Zoster (HZ) has not been so far adequately assessed in comparison with standard pharmacological treatment (ST) by a controlled trial design.

**Methods:**

Within the VZV Pescara study, pain was assessed in HZ patients on a Visual Analogue Scale (VAS) and by the McGill Pain Questionnaire (MPQ) both at the beginning and at the end of treatment. Response rates, mean changes in pain intensity, differences in total pain burden with an area-under-the-curve (AUC) method over a 1-year follow-up and differences in the incidence of Post-Herpetic Neuralgia (PHN) were evaluated.

**Results:**

One hundred and two patients were randomized to receive either AC (n = 52) or ST (n = 50) for 4 weeks. Groups were comparable regarding age, sex, pain intensity at presentation and missed antiviral prescription. Both interventions were largely effective. No significant differences were observed in response rates (81.6% vs 89.2%, p = 0.8), mean reduction of VAS (4.1 +/- 2.3 vs 4.9 +/- 1.9, p = 0.12) and MPQ scores (1.3 +/- 0.9 vs 1.3 +/- 0.9, p = 0.9), incidence of PHN after 3 months (48.4% vs 46.8%, p = 0.5), and mean AUC during follow-up (199 +/- 136 vs 173 +/- 141, p = 0.4). No serious treatment-related adverse event was observed in both groups.

**Conclusions:**

This controlled and randomized trial provides the first evidence of a potential role of AC for the treatment of acute herpetic pain.

**Trial registration:**

ChiCTR-TRC-10001146.

## Background

A significant proportion (18% to 41%) of patients suffering with Herpes Zoster (HZ) experience intense or very intense pain at presentation, frequently persisting after the healing of rash [1-4]. Both pain in the acute phase and chronic pain in post-herpetic neuralgia (PHN) may severely impact on quality of life and health care costs, interfering with physical, emotional and social functioning of affected patients [5-7]. Therefore, several investigations were carried out to identify effective strategies to control Zoster-related pain [8-11].

Current therapeutic options include the repeated administration of paracetamol and/or other NSAIDs for the immediate relief of pain [9,10], antiviral drugs, which accelerate the resolution of acute pain and may reduce the incidence of PHN [12-14], long-term gabapentinoids and local anaesthesia for selective nerve blockade in patients with intense or very intense pain at presentation [15-21]. Finally, opioid analgesics, including tramadol, possibly combined with other neuroactive agents, such as amitriptyline, have been used in the event of unresponsive pain [22-26]. In the most suffering patients, however, the control of pain is often unsatisfactory despite the administration of complex drug combinations, which also bear the potential of relevant metabolic effects [11,22].

In this context, the evidence that acupuncture may be efficacious in the treatment of neuropathic pain syndromes lead to the hypothesis of a potential beneficial effect of this procedure for acute HZ-related pain [27-29]. Therefore, several studies investigated the impact of acupuncture in patients with intense pain due to HZ or PHN, with controversial results [13,30-35]. None of them, however, was a randomized controlled trial for acute pain in HZ [13,36,37].

Here we report on a randomized, controlled trial, comparing the efficacy of acupuncture (AC) and standard pharmacological treatment (ST) in controlling intense or very intense acute pain in patients with HZ.

## Methods

### Study design and population

The present randomized clinical trial was nested within the "VZV Pescara Study", a cohort study aimed at evaluating the intensity of pain at presentation, pain persistence, overall pain burden and relative predictors in HZ patients [14]. Final approval of the study was granted by the Ethical Committee of the Local Healthcare Agency of Pescara in March, 2006. From May 2006 to April 2008, 41 General Practitioners (GPs) in the district of Pescara, Italy, the Infectious Diseases (ID) Unit, the Dermatology Unit, and Pain Management Clinic (PMC) of the Pescara General Hospital asked all incident cases of HZ to participate in the cohort study. All data were collected by the Infectious Disease Unit using a specifically created computerized network. All eligible patients granting signed informed consent were followed for 12 months. Patients enrolled in the cohort study with pain classified as intense or very intense on a five-degree semi-quantitative scale (no pain, mild, moderate, intense, very intense) were asked to participate in the present trial. At enrolment, a second specific informed consent was obtained, and the intensity of pain was re-assessed using a Visual Analogue Scale (VAS) [38,39]. If the intensity was ≥ 7 at VAS, participants were randomized to receive either standard pharmacological treatment (ST) or acupuncture (AC). The randomization process was carried out by the Statistical Unit and stratified by gender, age class (10 y) and pain intensity (VAS 7-8, and VAS 9-10). None of the investigators had any role in the allocation of patients.

### Intervention

**Figure 1:**
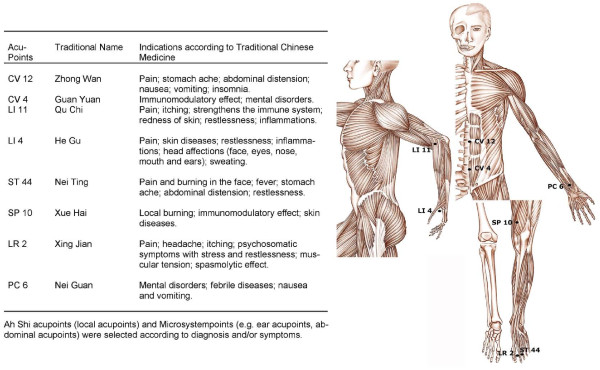
**Relevant Acupoints used in patients treated with Acupuncture**. (adapted from anatomic tables of Traditional Chinese Medicine, Associazione Gruppo Studio "Società e Salute", with permission).

In the AC arm, 8 sessions of Traditional Chinese Acupuncture were administered twice weekly by 2 experienced acupuncture physicians, members of the Italian School of Acupuncture and Traditional Chinese Medicine, Boulogne, Italy. Acupuncture basic points used are shown in Figure 1. In the ST arm, pregabalin was administered to all patients. The initial dose of 75 mg/d was gradually augmented based on patients' needs (maximum dose: 600 mg/daily, divided in 2 daily doses). In addition to the gabapentinoid, in the ST arm local anaesthesia was administered to all patients using 4-7 ml of chirocaine (1.5 mg/mL), either as intermittent peridural neural blockade in those with lumbar or sacral localization, or as intermittent perineural peripheral blockade in the remaining cases. Local anaesthesia was repeated every second day in patients complaining for yet uncontrolled pain, up to 5 administrations. Finally, in the ST arm transdermal buprenorphine (35-90 mcg/h) or oral oxycodone (50-400 mg daily) were prescribed to patients with very intense or refractory pain. Dosages of opioids were adjusted twice-weekly, in accordance with patients' complaints. Neither pregabalin, local anaesthesia nor opioids were allowed at any time for patients in the AC group; prescription of either drug was considered a protocol violation. For immediate pain relief, however, i.v. or oral paracetamol (250 to 1000 mg in accordance to both body weight and pain intensity) was allowed up to 3 times daily in both study arms. The duration planned for both treatments was 4 weeks.

### Outcomes

The main outcome of the study was the reduction in pain intensity between baseline and the end of the 4-week treatment, as measured by the reduction in the VAS score [38,39]. Secondary outcomes included: the reduction in the score of the McGill Pain Questionnaire (MPQ), which was administered by a psychologist for the multidimensional definition of pain in the individual patient [39-41]; the response rate (VAS score decrease by at least 2 points during treatment); the percentage of incident cases of PHN (defined as the presence of pain of any grade 3 months after enrolment - data obtained from the nesting cohort study); the total pain burden during the 12-month follow-up of the cohort study; finally, the incidence of treatment-related serious adverse events during treatment (grade 3 or higher according to the WHO scale) [42]. The total pain burden was quantified using an area-under-the-curve (AUC) method [14,43]. In brief, all patients in the cohort study were asked about pain severity at 1, 3, 6 and 12 months after enrolment, using the five-degree semi-quantitative scale described above. To obtain the AUC, measures of pain intensity were combined with pain duration, and each patient's AUC was calculated as the sum of all areas obtained by multiplying the average of 2 consecutive pain scores by the number of days between the scores.

### Sample size estimation

Because of the recent debate on non-inferiority trials [44], the study was conservatively planned with a superiority design. As mentioned above, the main outcome was the difference in the mean change of VAS scores from baseline to the end of the treatment between groups. According to previous studies [30-35] and inclusion criteria, the expected baseline mean VAS score was 8.0 ± 2.0 in both groups. The expected mean score at the end of the follow-up was 2.5 ± 2.0 in the acupuncture group; 4.0 ± 2.0 in the control group, with mean changes from baseline of 5.5 (2.0) and 4.0 (2.0), respectively. Using an unpaired t-test, and assuming an alpha-error = 0.05 and an expected withdrawal/dropout rate of 20%, a minimum of 34 subjects per group were requested to achieve a 80% statistical power.

### Data analysis

Kruskal-Wallis test was used to compare VAS, McGill and AUC mean scores across groups at each time point, as well as the mean change in each score during the follow-up. Chi-squared test was used to compare the VAS response rate and the prevalence of PHN between groups. Within each group, the differences in VAS and McGill scores between baseline and the end of the 4-week treatment were evaluated using paired t-test and confirmed through the Wilcoxon matched-pairs signed ranks test. A two-tailed p-value of 0.05 was considered significant for all analyses, which were carried out using Stata 10.1 (Stata Corp., College Station, TX, 2007).

## Results

### Characteristics of the sample

**Figure 2:**
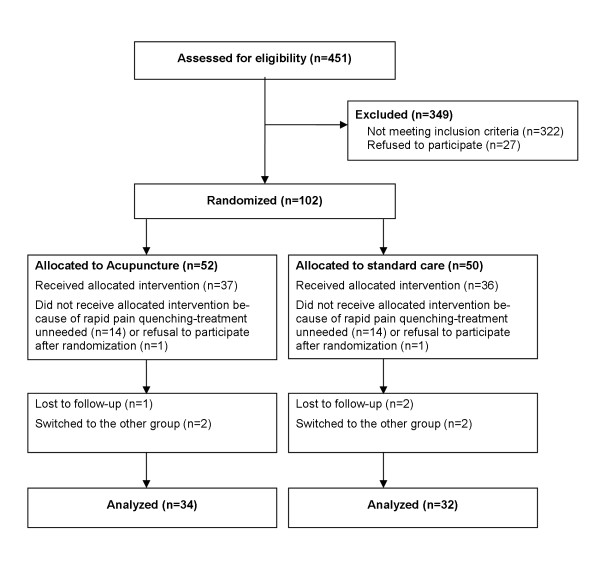
**Flow of participants through each stage of the trial**.

During the 2 years of enrolment in the VZV Pescara cohort study, 451 patients were clinically and/or microbiologically diagnosed as HZ [14]. Of them, 129 reported intense or very intense pain at presentation and were thus eligible for the present trial. Twenty-seven patients (21%) refused to participate, and 102 patients were randomized to receive either acupuncture (AC, n = 52) or standard therapy (ST, n = 50) (Figure 2). Many of the randomized patients, however, did not receive the allocated intervention, because of rapid pain quenching after enrolment (n = 13 in AC, n = 14 in ST), or for consent withdrawal after randomization (n = 1 in each group). Finally, 1 AC and 2 ST patients were lost to follow-up, and 2 patients in each group underwent protocol violations (Figure 2). The final analyses were thus based upon 34 patients in the AC group and 32 patients in the ST group (Figure 2).

In the initial sample of 102 randomized patients, no differences between groups were observed in mean age (63.2 ± 16.1 y in ST; 64.2 ± 14.7 y in AC), male gender (30.0% in ST and 35.5%, in AC), pain intensity at presentation (21.8% with very intense pain in ST; 23.6% in AC) and missed antiviral prescription (10.0% in ST and 15.4% in AC). Similarly, as shown in Table 1, demographic and clinical characteristics of the 66 patients entering the final analyses were well balanced between groups. Finally, no differences were observed between the 66 patients in the final sample and the 27 patients refusing to participate (mean age 64.2 ± 16.0 y, males 18.5%, 17.4% with very intense pain). By contrast, and expectedly, the 28 patients excluded after randomization because of rapid pain reduction were significantly younger (mean age 56.6 ± 19.8 y, p < 0.01) and with less severe pain at presentation (7.1% with very intense pain, p = 0.04).

**Table 1:** Demographic and clinical characteristics of treated patients, by group.

Characteristics	Standard Therapy (n = 32)	Acupuncture (n = 34)	p*
Male gender, %	40.6	32.3	0.5
Mean age in years (SD)	65.5 (12.8)	67.1 (12.8)	0.6
Current smoking, %	37.5	38.1	0.8
High-school or higher educational level, %	28.1	35.3	0.5
Depression (clinical diagnosis), %	0.0	5.9	0.2
HIV-positive, %	0.0	0.0	0.9
Missed antiviral prescription, %	15.6	17.6	0.8
Very intense pain at presentation, %	30.0	29.0	0.9
Vesicles (>50), %	35.0	48.4	0.3
Trauma at the site of VZV up to 6 m before onset, %	28.1	39.4	0.3
Surgical intervention at the site of rash up to 6 months before onset, %	59.4	55.9	0.8
*Site of lesions*			
Facial	9.4	23.5	0.12
Cervical	6.3	8.8	0.7
Thoracic	53.1	44.1	0.5
Lumbar	31.2	23.5	0.8
*Extension of lesions*			
Subdermatomerical	28.1	26.5	0.9
Dermatomerical	59.4	67.6	0.5
Multidermatomerical	12.5	5.9	0.3
*Antiviral therapy*			
Acyclovir	40.6	32.4	0.5
Famcyclovir	0	5.9	0.2
Valacyclovir	31.3	29.4	0.9
Brivudin	9.4	11.8	0.8
Other antiviral	3.1	2.9	0.9
Missed antiviral prescription	15.6	17.6	0.8

* Fisher's exact test for categorical variables, t-test for continuous variables (age).

### Efficacy of acupuncture and standard analgesic therapy

As shown in Table 2, the 2 groups were also similar at baseline as to the mean VAS and McGill scores (p = 0.6, p = 0.8, respectively). Both interventions were largely effective: the mean percentage of VAS score reduction from baseline to week 4 was of 51% in the ST group (mean reduction in VAS score = 4.12 ± 2.3), and 62% in the AC group (mean reduction in VAS score = 4.85 ± 1.9); the mean percentage of McGill score reduction was 57% and 56%, respectively. Indeed, a highly significant improvement from baseline to the end of the follow-up was observed in each group for both pain scores, and correspondent p-values were thus not reported in the table (all p < 0.001).

**Table 2:** Comparison of the outcomes of treatments under evaluation.

Outcomes	Standard Therapy (n = 32)	Acupuncture (n = 34)	p*
***Primary outcomes ***			
*VAS*			
Mean VAS score at baseline (SD)	8.02 (1.69)	7.81 (1.48)	0.6
Mean VAS score after therapy (SD)	3.89 (2.38)	2.96 (1.84)	0.08
Mean change in VAS score (SD)	4.12 (2.29)	4.85 (1.87)	0.12
Response rate (>=2 VAS units decrease), %	81.6	89.2	0.8
***Secondary outcomes***			
*McGill score*			
Mean McGill score at baseline (SD)	2.32 (1.05)	2.38 (1.12)	0.8
Mean McGill score after therapy (SD)	0.99 (0.69)	0.99 (0.73)	0.9
Mean change in McGill score (SD)	1.32 (0.85)	1.33 (0.88)	0.9
Post-herpetic neuralgia at 3 months, %	48.4	46.8	0.9
Post-herpetic neuralgia at 6 months, %	33.3	29.0	0.7
Post-herpetic neuralgia at 12 months, %	10.3	3.6	0.3
Mean AUC during follow-up (SD)	199(136)	173(141)	0.5

VAS = Visual Analogical Scale. AUC = Area under the curve (see text for details).

* Kruskal-Wallis test was used to compare VAS, McGill and AUC mean scores across groups at each time point, as well as the mean change in each score during the follow-up. Chi-squared test was used to compare the VAS response rate and the prevalence of PHN at each time point across groups. A paired t-test was used to compare baseline vs end of therapy mean values of VAS and McGill scores, within each group. All such differences were highly significant (p < 0.001) and were thus omitted in the table to avoid redundancy.

### Comparison between acupuncture and standard analgesic therapy

No significant differences were observed between the 2 therapeutic approaches in any of the outcomes under consideration (Table 2). Neither the mean reduction in VAS score (p = 0.12) or in McGill score (p = 0.9), nor the response rate (p = 0.8), nor the incidence of PHN after 3 months (p = 0.5), nor the mean AUC (total pain burden) during follow-up (p = 0.4) were significantly different across groups.

As to safety, no serious adverse events (grade 3-4 according to WHO safety scale) related to both treatments were observed in the 2 groups. In addition, none of the patients were hospitalized or died during the 4 week treatment. Finally, none of them discontinued treatment due to drug-related complications or procedure-related discomfort.

## Discussion

Lack of proper efficacy evaluations, adequate statistical power and rational design of the previous studies assessing the role of Acupuncture for Zoster related acute pain is acknowledged [36,37]. Coghlan reported in 1992 the treatment of a small case series of patients with pain caused either by acute HZ or by PHN, including 4 patients each. Electro-acupuncture alone was used and reported as generally effective, endorsing the opportunity for an exploratory trial [30]. More recently, Ni et al. reported the treatment of 48 cases of HZ by nerve stem puncturing. Among these, all of the 16 cases treated within 1 week from pain onset were reported as cured, in comparison with only 5 (15.6%) among the 32 cases whose pain lasted over 1 week before treatment [33]. This study was based on a consecutive case series and lacked of any control arm [33]. He et al. [32] described 60 cases of senile HZ treated by encircled acupuncture plus valacyclovir (300 mg twice daily orally for 10 days) versus valacyclovir only. The authors report that patients were divided into 2 well balanced treatment groups, not mentioning any randomization process; no alternative analgesic treatment was administered to the control group [32]. The only significantly different outcome between groups was the duration of acute pain (3.8 days for combined treatment vs 5.5 days for valacyclovir only); furthermore, a lower incidence of pain persistence during follow-up was reported for patients undergoing combined treatment (26.7 vs 53.3%), although no definition was provided neither for PHN nor for the duration of follow-up [32]. Therefore, to our knowledge, this is the first randomized clinical trial assessing the efficacy of acupuncture for the control of HZ-related intense or very intense acute pain as well as for the prevention of PHN during follow-up [30,32,33,36,37]. We found that both AC and ST, associated with the prescription of antivirals, were efficacious in controlling intense or very intense pain. Importantly, the degree of pain control provided by AC was not significantly different from that of ST.

Pain intensity was evaluated by the most frequently used methods in VZV-related pain studies: VAS, to obtain an easy quantification of pain intensity [38,39]; McGill Pain Questionnaire to qualifying pain [39-41]. We found no significant difference both in VAS and McGill scores after 4 weeks of treatment between AC and ST. Since pain intensity at rash onset is widely reported as the best predictor of PHN [6,45-48], having selected for patients with intense or very intense pain we also evaluated the incidence of PHN at 3, 6 and 12 months after rash onset, as well as the total pain burden during follow-up in the 2 study arms. Under the assumption that acupuncture may have an immune modulating activity [49-52], evaluating these endpoint might have revealed an ability of this medical tool to influence the rate of pain persistence and relapses. The incidence of PHN at 3, 6 and 12 months, as well as the mean AUC were overlapping in the 2 arms (Table 2). The limited dimensions of our population do not allow, however, to conclusively exclude, although unlikely, this hypothesis.

It should be noted that the stratification for pain resulted in a perfect match of patients with very intense pain at presentation, which could have otherwise remarkably influenced the validity of our results for all endpoints. Furthermore, the percentage of patients which did not receive antiviral therapy was identical in the 2 arms (15.6% and 17.6%, respectively), protecting our trial from another possible confounding factor, that is a difference in potential control of acute HZ pain by antiviral therapy between groups [1,53].

This study has some limitations, however, that must be considered in interpreting results. First, we decided to measure pain intensity both by VAS and McGill scales at study entrance and after 4 weeks of treatment, that is when the investigator felt that his intervention for the control of acute pain was complete. Clearly, it would have been interesting to measure ongoing pain control at intermediate time points, to better appreciate minor differences between the investigational arms. This was not feasible, however, due to study budget constraints; in any case, most of the other investigations assessing the efficacy of experimental treatments for pain control were built with very similar or identical designs [54,55].

Second, our trial was based upon a relatively small sample of treated patients (n = 66). However, 41 GPs and 3 hospital units were committed to recruit HZ patients for 2 years, and more than 400 HZ patients had to be enrolled to identify 129 patients with intense or very intense pain at presentation (those with the highest need of improvements in pain control). In addition, a relatively large number of the eligible patients (n = 27) refused to enter the trial. However, this was probably unavoidable because of distrust or fear of an unusual technique such as acupuncture. Notably, no differences in age, gender and pain intensity were observed between these patients and those included in our analyses. Finally, 28 patients were excluded immediately after randomization because of rapid pain reduction. However, it would clearly have been unethical to enforce these patients in a strong analgesic therapy as well as acupuncture. Notably, and expectedly, the latter patients were younger and had very intense pain in only 2 cases. This is the reason why it would have had very little sense to use an intention-to-treat analysis, which is usually the gold standard analysis for RCTs [56], in this case. In other terms, the 28 patients with rapid pain reduction represent a separate group of patients, not needing long term analgesic therapy; they therefore may not carry the potential for selection bias. As a final remark on sample size, it should be noted that the results of the 2 interventions were very similar and, using a one-tailed non-inferiority approach, the statistical power of our analysis is acceptable for all outcomes, ranging from a minimum of 81% for the change in VAS score, up to 86% for the change in McGill score.

A third limitation is represented by the lack of both a control arm with short acting analgesics only, and a control arm with mock acupuncture [57-59]. This was beyond our scope because of study budget constraints. However, as the access to alternative drugs and complementary medicine techniques for acute pain control is on the rise in our area and elsewhere in the world, we felt that the design of this nested trial, investigating "real life" differences in therapeutic potential between ST and AC as presently used in severely ill HZ patients, might still add to the knowledge on the analgesic efficacy of acupuncture. Indeed, proper stratification of patients between arms rather than blinding might have influenced the assessment of the efficacy of either intervention, that is taking into account - when distributing patients between arms - the main factors that may influence the efficacy of either intervention, such as age, pain intensity and missed antiviral prescription. These variables were indeed well-matched in our study population. A direct comparison between ST and AC for acute pain in HZ is the object of another three-armed, partially blinded randomized trial, known as ACUZoster, whose design was recently published and which is still ongoing [60].

## Conclusions

With these caveats, our study provides the first evidence from a randomized controlled design of a potential role of acupuncture in the treatment of acute herpetic pain. Patients with intense or very intense pain at presentation showed a significant and similar degree of pain relief using acupuncture and standard pharmacological therapy. Also, no differences between treatments were observed in the incidence of severe adverse events. Given that patients treated with acupuncture carry a lower risk of cumulative drug toxicity, if these findings will be confirmed by the ensuing ACUZoster trial and/or by other investigations, acupuncture might be appropriately considered among the available therapeutic options for the control of severe acute HZ related pain.

## Abbreviations

AC: Acupuncture; ST: Standard Pharmacological Treatment; VAS: Visual Analogue Scale; MPQ: McGill Pain Questionnaire; AUC: Area Under the Curve; PHN: Post Herpetic Neuralgia; HZ: Herpes Zoster; GPs: General Practitioners; ID: Infectious Disease; PMC: Pain Management Clinic.

## Competing interests

The authors declare that they have no competing interests.

## Authors' contributions

GP, LP, TU, CR, CG CDA and MT conceived and coordinated the study; GP, LM, LP, CDA and CG designed the trial. LM, LP, GP, EP, AC, CDA and CG interpreted and discussed the results of the trial; LM, GP and EP performed the statistical analyses; CR prescribed and performed the standard pharmacological analgesic treatments; LP and GC performed acupuncture treatments; SDP, AC and GPl administered and corrected McGill Pain questionnaire; PMT and SL randomized and tracked all patients; TU, GP, MT, AC and GPl followed the clinical status of patients under observation; SL, CG and PMT cared for the online database; GP, TU, MT, EP, LM, CG and CDA wrote and revised the manuscript. All authors read and approved the final manuscript.

## Pre-publication history

The pre-publication history for this paper can be accessed here:

http://www.biomedcentral.com/1472-6882/11/46/prepub
